# Effects of light intensity and reduction of starter diet digestible lysine and metabolizable energy on broiler chicken growth performance, breast meat yield, and meat quality defects

**DOI:** 10.1016/j.psj.2023.103222

**Published:** 2023-10-21

**Authors:** Joshua J. Flees, A. Jacob Keel, Caroline R. Gregg, Charles W. Starkey, Jessica D. Starkey

**Affiliations:** Department of Poultry Science, Auburn University, Auburn, AL, 36849, USA

**Keywords:** light intensity, starter dietary nutrient restriction, digestible lysine reduction, metabolizable energy reduction, Wooden Breast, broiler growth performance

## Abstract

The etiology of Wooden Breast (**WB**) is unknown; therefore, it is difficult to produce broiler flocks with similar proportions of WB-affected and unaffected birds. Because WB has been detected as early as 15 d posthatch, the objective of this randomized complete block experiment with a 2 × 2 factorial treatment arrangement was to determine whether combining the effects of light intensity (**LI**) and early nutrient reduction strategies could reliably produce WB-affected and normal broilers to further investigate the physiological mechanisms underlying WB. On day of hatch, male, Ross 708 × Yield Plus broilers (n = 384; 16 birds per pen; 3 replicate blocks) were randomly allotted to floor pens in the same facility and exposed to either 2 (**LOWLI**) or 30 (**HIGHLI**) lux of light from d 0 to 35. Birds were fed either a commercial starter diet (**CON**) or the CON diet with a 10% reduction in both ME and digestible lysine (**dLys; RED**) from d 0 to 14 and then a common grower diet from d 15 to 35. Broiler growth performance, breast yield, and incidence and severity of WB and White Striping (**WS**) were assessed. Data were analyzed as a 2-way ANOVA with SAS PROC GLIMMIX and means separated at *P* < 0.05 with PDIFF. No interaction among LI and diet was observed (*P* > 0.05). Broilers reared with HIGHLI were heavier on d 35 and consumed more feed in all phases compared with broilers reared under LOWLI (*P* ≤ 0.0096). Broilers reared under LOWLI gained less BW from d 15 to 35 and d 0 to 35 compared with broilers reared under HIGHLI (*P* = 0.0073). Broilers fed the RED starter diet consumed more feed and had higher FCR from d 0 to 14 compared with broilers fed the CON diet (*P* ≤ 0.0012). In conclusion, combining reductions in LI and starter diet ME and dLys did not produce the hypothesized reductions in breast yield and incidence and severity of WB or WS.

## INTRODUCTION

A rise in the consumption of poultry meat over the past several decades has led to more intensive selection pressure placed on increasing growth rate while improving feed efficiency of broiler chickens to keep up with consumer demand (Zuidhof et al., 2014). Unfortunately, the improvements in growth rate, breast muscle size, and breast meat yield have led to an increase in the incidence of the meat quality defect known as Wooden Breast (**WB**) in the *Pectoralis major* (breast) muscle of broiler chickens. The WB meat quality defect is phenotypically characterized by a pale color, bulging appearance, loss of elasticity, and abnormal hardness. Further research into the defect revealed a decrease in marinade uptake and water holding capacity, an increase in cooking losses and pH, and an undesirably firm texture in WB-affected meat (Mudalal et al., 2015; Chatterjee et al., 2016; Soglia et al., 2016; Wang et al., 2023) which can negatively impact consumer acceptance (de Almeida Assunção et al., 2020) leading to high economic losses. From a physiological standpoint, degradation of myofibers and infiltration of connective tissue have been reported in broilers severely affected with WB (Sihvo et al., 2014; Kawasaki et al., 2018; Velleman, 2020).

Due to an unknown etiology, many researchers have investigated nutritional strategies (Meloche et al., 2016b; Bodle et al., 2018; Meloche et al., 2018d; Zampiga et al., 2019; Wang et al., 2020; Kuttappan et al., 2021), nutrient restriction (Meloche et al., 2016a; Meloche et al., 2018b,c; Iwasaki et al., 2021; Vieira et al., 2021), and feed restriction strategies (Livingston et al., 2019; Simões et al., 2020) to reduce the incidence of WB while attempting to maintain optimal growth performance. While WB incidence can be highly variable among modern broiler flocks, previous research indicates that the incidence of WB can be decreased by reducing concentrations of digestible lysine (**dLys**) critical for breast muscle growth during specific phases of growth (Cruz et al., 2017; Meloche et al., 2018a,c). By modulating dLys content in grower phase diets, researchers have been able to produce broilers within the same flock that are differently-affected by WB to be used as a model to further study the etiology (Meloche et al., 2018a; Ferreira et al., 2020). Yet, WB can be detected as early as d 15 and often before transitioning broilers to the grower feeding phase of production (Avila et al., 2022). Thus, it is possible that earlier implementation of a nutritional strategy to slow broiler growth rate during the starter phase of production could be effective in reducing incidence and severity of WB. Other management strategies such as high light intensity (**LI**) has been shown to decrease growth performance due to an increase in broiler activity (Rault et al., 2017); however, the combination of management and nutritional strategies to slow early broiler growth rate with the goal of reducing the incidence and severity of WB have yet to be investigated. Therefore, the objective of this study was to utilize dLys and ME nutrient reductions in starter diets while rearing broilers under different LI to understand their impacts on broiler growth performance and the incidence and severity of WB to find a strategy to slow broiler growth and produce broilers within the same flock differently affected by WB.

## MATERIALS AND METHODS

All animal experiments were approved by the Auburn University Institutional Animal Care and Use Committee (protocol 2017-3213).

### Broiler Husbandry

Day-old, male Ross 708 × Yield Plus broiler chicks (n = 192) were obtained from a commercial hatchery and randomly distributed into 12 floor pens (16 birds per pen) within the same light-controlled facility. Each pen was equipped with a hanging feeder, nipple drinker water line, and new pine shavings. Birds consumed feed and water on an ad libitum basis with feeder and water line heights adjusted based on bird height. At placement, ambient temperature was set at 32.5°C and was gradually decreased to maintain bird comfort until a final set point of 20°C was achieved.

### Experimental Design

This experiment was a randomized complete block design with a 2 × 2 factorial treatment structure. The 12 pens were divided into 3 replicate blocks of 4 pens with each pen assigned to 1 of the 4 treatment groups. Broilers were reared under either 30 lux of LI (**HIGHLI**), or 2 lux of LI (**LOWLI**) adjusted at litter level using a photometric sensor with National Institute of Standards and Technology-traceable calibration (403125, Extech Instruments, Waltham, MA) for each intensity adjustment. The LOWLI intensity of 2 lux was chosen as it was below the typical broiler industry LI and was the lowest LI we could achieve in the bird chamber utilized for this experiment. The LI was maintained from d 0 to 6 with a 23 h of light and 1 h of dark photoperiod, and from d 7 to 35 with an 18 h of light and 6 h of dark photoperiod. Broilers reared under either LOWLI or HIGHLI were fed either a commercial starter diet as the control diet (**CON**) or a nutrient-reduced diet (**RED**) which was the CON diet formulated with a targeted 10% reduction in dLys and ME. Birds were fed either CON or RED starter diets from d 0 to 14 and then all were fed a common commercial grower diet from d 15 to 35. The starter diets were provided to birds in crumble form while the grower diet was provided in pellet form. Diet formulations and calculated nutrient composition of the starter and grower diets are shown in Table 1.

**Table 1:** Ingredient and calculated nutrient composition of starter and grower diets.

	Starter diet (d 0–14)		Grower diet (d 15–35)
	CON	RED	
Ingredients, %			
Corn	61.67	55.89	67.90
Soybean meal	33.00	30.00	27.74
Soybean oil^1^	1.96	-	1.20
Wheat middlings	-	2.29	-
Dried distiller's grains with solubles	-	5.00	-
Sand	-	3.50	-
Dicalcium phosphate	0.65	0.65	0.65
Limestone	1.20	1.20	1.22
Salt	0.26	0.26	0.26
L-Lysine hydrochloride, 98%	0.35	0.29	0.28
DL-methionine	0.37	0.37	0.29
L-threonine	0.25	0.27	0.19
Phytase^2^	0.03	0.03	0.03
Vitamin premix^3^	0.10	0.10	0.10
Trace mineral premix^4^	0.10	0.10	0.10
Calcium chloride, 60%	0.05	0.05	-
Calculated Analysis			
Crude protein, %	20.6	20.5	18.3
Metabolizable energy, kcal/kg	2,866	2,579	2,867
Digestible lysine, %	1.280	1.152	1.090
Lysine, %	1.411	1.310	1.213
Digestible methionine, %	0.650	0.650	0.550
Methionine	0.689	0.693	0.586
Calcium, %	0.967	0.970	0.960
Available phosphorus, %	0.523	0.535	0.519

1 Soybean oil was added in the mixer.

2 Optiphos 6000.

3 Vitamin premix provided the following per kilogram of diet: Vitamin A (Vitamin A acetate), 9,370 IU; Vitamin D (cholecalciferol), 3,300 IU; Vitamin E (DL-alpha tocopheryl acetate), 33 IU; menadione (menadionesodium bisulfate complex), 2 mg; Vitamin B12 (cyanocobalamin), 0.02 mg; folacin (folic acid), 1.3 mg: D-pantothenic acid (calcium pantothenate), 15 mg; riboflavin (riboflavin), 11 mg; niacin (niacinamide), 44 mg; thiamin (thiamin mononitrate), 2.7 mg; D-biotin (biotin), 0.09 mg; pyridoxine (pyridoxine hydrochloride), 3.8 mg.

4 Mineral premix provides the following per kg of diet: Mn (manganese sulfate), 120 mg; Zn (zinc sulfate), 100 mg; Fe (iron sulfate monohydrate), 30 mg; Cu (tri-basic copper chloride), 8 mg; I (stabilized ethylenedi-amine dihydriodide), 1.4 mg; Se (sodium selenite), 0.3 mg.

### Measurements

Broilers were individually weighed on day of placement (d 0), at the end of the starter phase (d 14), and at the end of the experiment (d 35). All feeders were individually weighed at the end of the starter phase and end of the trial for calculation of FI. Mortalities were collected daily and weighed to calculate mortality-corrected FI, BWG, and FCR. On d 36, 10 birds reared under HIGHLI fed the CON diet, 15 birds reared under HIGLI fed the RED diet, 11 birds reared under LOWLI fed the CON diet, and 13 birds reared under LOWLI fed the RED diet (n = 50) were euthanized by cervical dislocation, and the whole breast muscle was removed, weighed, and evaluated by 1 trained and experienced individual for both WB and White Striping (**WS**). A 4-point scale (0 = normal; 1 = mild; 2 = moderate; 3 = severe) for WB and WS was used. WB and WS scores of 0 were assigned to fillets considered normal and that exhibited complete absence of the defect. Score 1 breasts were mildly affected (up to 25% of the fillet), Score 2 breasts were moderately affected (26%–50% of the fillet), and Score 3 fillets were severely affected (more than 50% of the fillet) as previously described by Tejeda et al. (2021).

### Statistical Analysis

Performance data were analyzed as a randomized complete block design with a 2 × 2 treatment structure using pen location as the blocking factor and pen as the experimental unit. Light intensity (HIGHLI vs. LOWLI), starter diet (CON vs. RED), and their interactions served as the fixed effects in the model statement. Data were subjected to a 2-way ANOVA using PROC GLIMMIX of SAS 9.4 (SAS Institute, Cary, NC) using the following model:

$$Y_{ij}=\;\mu +\;\pi_{i}\;+\;\alpha_{j}+\;\pi \alpha_{ij}+\;\varepsilon_{ij}$$

where µ is the grand mean; *π_i_* are the independently normally distributed random block effects with mean 0 and variance *σ*^2^_a_; *α_j_* are the mean factor levels analogous to the *j*th treatment such that ∑αj=0; *πα_ij_* are the interactions among the fixed factor levels and the treatments with the mean 0 and variance *σ*^2^_b_; and finally, *ε_ij_* are independently and identically distributed random errors with mean 0 and variance *σ*. The Satterthwaite adjustment was used to correct the degrees of freedom. Proportions of affected fillets in each scoring category were analyzed by PROC GLIMMIX (SAS Institute) using the events/experiments syntax with a binomial distribution and R-side covariance structure. Where applicable, residuals were visually assessed to ensure normality and non-normal data were transformed prior to analysis. For all hypothesis tests, least square means were separated using the PDIFF option of SAS and means were declared different when *P* ≤ 0.05 and tendencies declared when 0.0501 < *P* ≤ 0.10. Statistical analysis revealed no interaction among LI and starter diet for any variables; therefore, only the main effects of LI and starter diet are presented and discussed.

## RESULTS AND DISCUSSION

### Effect of Light Intensity on Broiler Growth Performance, Breast Meat Yield, and Incidence and Severity of WB and White Striping

Birds reared under HIGHLI (30 lux) were heavier compared with broilers reared under LOWLI (2 lux) on d 35 (Table 2; *P* = 0.0386). During the starter (d 0–14 period, birds reared under HIGHLI consumed more feed compared with birds reared under LOWLI (Table 2; *P* = 0.0153). Broilers tended to gain more BW when reared under HIGHLI compared with birds reared under LOWLI during the grower (d 15–35) phase (Table 2; *P* = 0.0780). These results indicate that rearing birds under a 30 vs. 2 lux LI may improve growth performance during the grower phase and result in heavier birds at d 35, which is in agreement with previous literature. A study revealed that birds reared under 30 lux of light had a 1.9% decrease in BW compared with birds reared under 15 lux of light (Velo and Ceular, 2017). Results from Rault and others revealed similar findings where birds reared under 20 lux of light were lighter at 46 d of age compared with birds reared under 5 lux of light (Rault et al., 2017). Furthermore, other published data revealed that birds reared under 0.1 foot-candles (1 lux) of light were heavier and consumed more feed compared with birds reared under 15 foot-candles (161 lux) of light (Lien et al., 2008). At the lower end of experimental light intensities, it was observed that increasing intensity from 0.5 to 5 lux can improve growth performance (Deep et al., 2013), and this range in intensity is more similar to the 2 lux utilized in this present study. More recently, rearing broilers for 35 d at a range of different LI (5, 20, 35, and 50 lux) did not impact BWG, FI, or FCR (Kim et al., 2022). In fact, these authors observed an increase in serum corticosterone in birds reared at 5 lux compared with all other LI, suggesting that the lowest LI resulted in higher stress which may help explain the results of the present experiment. Raccoursier et al. (2019) designed an experiment to evaluate broiler preference for various light intensities. They observed greater feed consumption in the area of the pen maintained at 20 lux compared with 5 lux. This may suggest that birds in the present study were stimulated to consume more feed in the HIGHLI environment, resulting in heavier BW at the end of the 35-d study.

**Table 2:** Effect of light intensity on broiler growth performance.

	Light intensity^2^			
Variable^1^	HIGHLI (30 lux)	LOWLI (2 lux)	SEM^3^	*P*-value
d 0 BW, g	40.3	40.3	0.2	1.000
d 14 BW, g	493.5	484.3	8.4	0.4613
d 35 BW, g	2,517.2^a^	2,430.3^b^	24.8	0.0386
d 0 to 14 MCFI, g	489.0^a^	465.3^b^	5.5	0.0153
d 0 to 14 MCBWG, g	397.2	387.0	7.3	0.3533
d 0 to 14 MCFCR	1.2331	1.2028	0.0117	0.2298
d 15 to 35 MCFI, g	2,889.3	2,775.2	47.9	0.1303
d 15 to 35 MCBWG, g	1,984.2^x^	1,892.3^y^	32.1	0.0780
d 15 to 35 MCFCR	1.5643	1.6129	0.0585	0.5733
d 0 to 35 MCFI, g	2,858.2	2,750.5	64.3	0.2704
d 0 to 35 MCBWG, g	2,046.0	1,969.8	43.1	0.2465
d 0 to 35 MCFCR	1.3971	1.3959	0.0048	0.8628

1 On an individual bird basis: BW: body weight; MCBWG: mortality corrected body weight gain; MCFCR: mortality corrected feed conversion ratio (MCFI:MCBWG); MCFI: mortality corrected feed intake.

2 HIGHLI: high light intensity (30 lux; n = 6 replicate pens); LOWLI: low light intensity (2 lux; n = 6 replicate pens).

3 SEM: largest standard error of the least square means.

ab Means within a row with different superscripts differ at *P* ≤ 0.05.

x,y Means within a row with different superscripts differ at 0.0501 < *P* ≤ 0.10.

**Figure 1:**
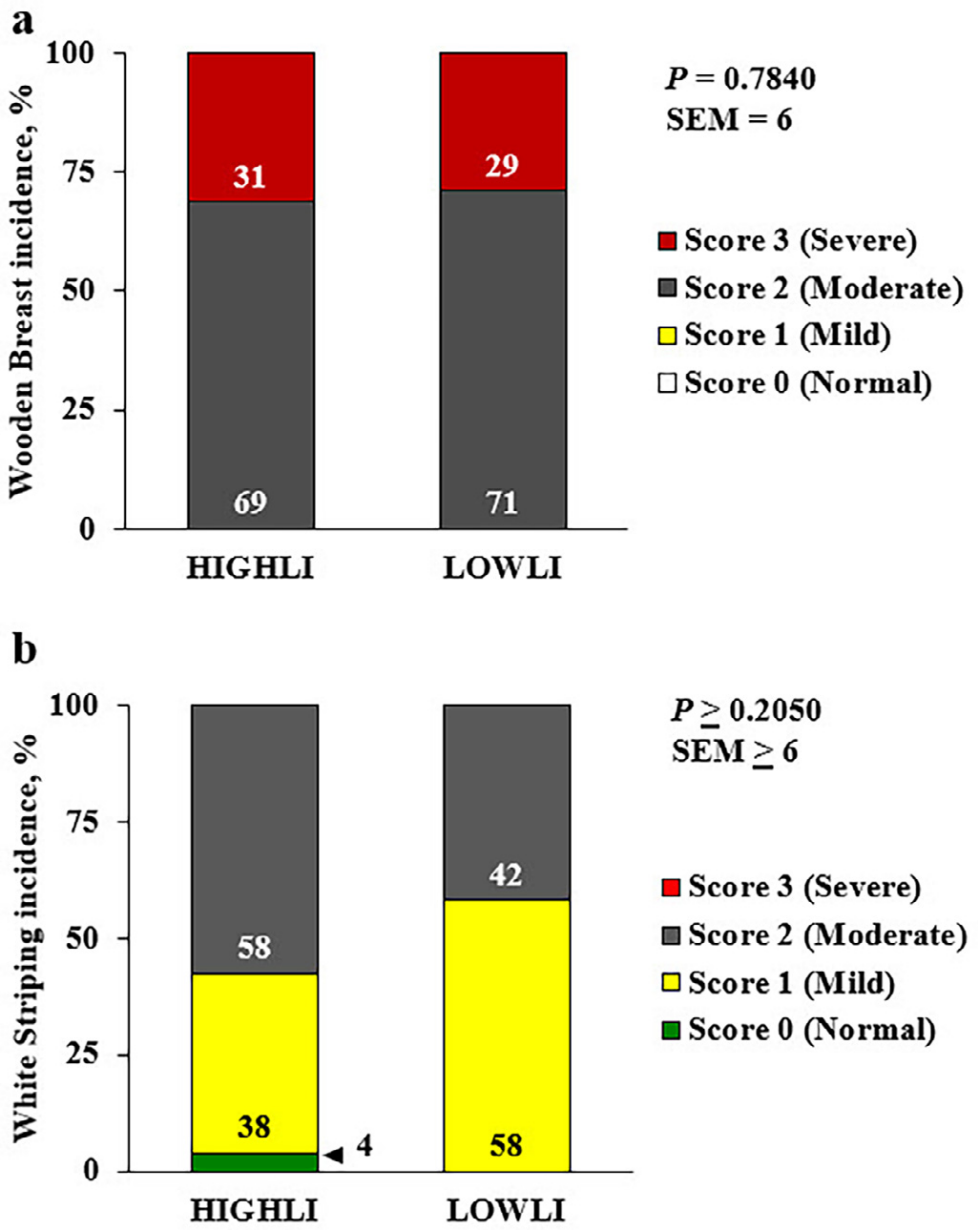
Effect of light intensity on the incidence and severity of Wooden Breast (Panel A) and White Striping (Panel B) in broiler chickens at 36 d of age. Proportions of normal (0; green), mild (1; yellow), moderate (2; gray), and severe (3; red) scores for Wooden Breast and White Striping observed among male, Ross 708 × Yield Plus broiler chickens at 36 d of age. Broilers were reared under 30 lux (HIGLI; n = 26 sampled birds) or 2 lux (LOWLI; n = 24 sampled birds) of light at litter level with photoperiods of 23 h of light and 1 h of darkness from d 0 to 6, and from d 7 to 35, 18 h of light and 6 h of darkness. SEM, standard error of the mean.

While changes were observed in broiler growth performance, there were no differences observed on breast weight (*P* = 0.9279), breast meat yield (*P* = 0.8828), mean score of WB (*P* = 0.8619), or the mean WS score (*P* = 0.9115; Table 3) when birds were reared under HIGHLI or LOWLI. Similarly, the incidence and severity of WB and WS were unchanged among LI treatment groups (Figures 1A and 1B; *P* > 0.05). Similar to the growth performance data, this data is in disagreement with other published work where a low lux LI at 0.1 foot-candles (1 lux) of light increased lean carcass, total breast weight, tender yield, leg yield, and wing yield (Lien et al., 2008), and 5 lux of light increased breast meat yield (Deep et al., 2013). While this current study is the first (to our knowledge) to study the effect of LI on both the incidence and severity of WB and WS, the incidence (% affected) and severity (Score 0, 1, 2, or 3) were similar among LI treatments (*P* ≥ 0.2050), though it is noteworthy to point out that 100% of the broilers were at least moderately affected with the WB phenotype (Score 2 or 3), and neither treatment produced any normal (Score 0) or mildly (Score 1) WB-affected fillets were observed in this flock on d 35 (Figure 1A). In terms of WS incidence and severity, the LOWLI treatment resulted in the complete absence of normal (Score 0) fillets, 58% incidence of Score 1 or mildly affected, and 42% incidence of Score 2 moderately affected fillets, while neither treatment produced any severely WS-affected (Score 3) fillets (Figure 1B). Overall, the lack of statistical differences among the treatment groups may indicate that managing broilers with varying LI up to 35 d of age may not impact the development of the WB and WS meat quality defects even though the HIGHLI broilers were heavier when harvested and scored.

**Table 3:** Effect of light intensity on broiler breast weight, breast yield, and mean Wooden Breast and White Striping scores on d 36.

	Light intensity^2^			
Variable^1^	HIGHLI (30 lux)	LOWLI (2 lux)	SEM^3^	*P*-value
Breast weight, g	485	483	13	0.9279
Breast yield, %	18	18	0.3	0.8828
Mean WB score	2.3	2.3	0.1	0.8619
Mean WS score	1.5	1.5	0.1	0.9115

1 On an individual bird basis: Breast weight = total breast muscle weight grams; breast yield = breast meat weight as a percentage of body weight; mean WB score = mean Wooden Breast score; mean WS score = mean White Striping score.

2 HIGHLI: high light intensity (30 lux; n = 26 sampled birds); LOWLI: low light intensity (2 lux; n = 24 sampled birds).

3 SEM: largest standard error of the least square means.

### The Effect of Starter Diet Nutrient Reduction on Broiler Growth Performance, Breast Meat Yield, and Incidence and Severity of Wooden Breast and White Striping

On d 0, chicks assigned to the CON treatment were lighter (39.8 vs. 40.8 g; *P* = 0.0028) than those on the RED treatment, while at d 14 bird BW was similar among treatments (*P* = 0.4158), yet on d 35 the birds fed the RED diet tended to be heavier than those fed the CON diet (Table 4; *P* = 0.0708). Birds that were fed the RED starter diet with a targeted 10% reduction in dLys and ME consumed more feed (*P* = 0.0118) and had higher FCR (*P* = 0.0380) from d 0 to 14 (Table 4). Though they consumed similar amounts of feed during the grower phase (*P* = 0.1404) and overall study period (*P* = 0.3862), the RED birds were heavier compared with the CON-fed birds on d 35 (2,510.3 vs. 2,437.2 g; *P* = 0.0708) due to the nearly 100 g of compensatory gain (1,987.0 vs. 1,889.5 g of BWG) they experienced during the grower (d 15–35) phase when they were allowed to consume the CON grower diet (Table 4).

**Table 4:** Effect of starter diet nutrient reduction on broiler growth performance.

	Starter diet^2^			
Variable^1^	CON	RED (10% ↓ dLys and ME)	SEM^3^	*P*-value
d 0 BW, g	39.8^b^	40.8^a^	0.2	0.0028
d 14 BW, g	483.8	494.0	8.4	0.4158
d 35 BW, g	2,437.2^y^	2,510.3^x^	24.8	0.0708
d 0 to 14 MCFI, g	464.7^b^	489.7^a^	5.5	0.0118
d 0 to 14 MCBWG, g	391.0	393.2	7.3	0.8389
d 0 to 14 MCFCR	1.1891^b^	1.2469^a^	0.0117	0.0380
d 15 to 35 MCFI, g	2,776.8	2,887.7	47.9	0.1404
d 15 to 35 MCBWG, g	1,889.5^y^	1,987.0^x^	32.1	0.0643
d 15 to 35 MCFCR	1.6520	1.5252	0.0585	0.1640
d 0 to 35 MCFI, g	2,762.7	2,846.0	64.3	0.3862
d 0 to 35 MCBWG, g	1,980.8	2,035.0	43.1	0.3999
d 0 to 35 MCFCR	1.3946	1.3984	0.0048	0.5910

1 On an individual bird basis: BW: body weight; MCBWG: mortality corrected body weight gain; MCFCR: mortality corrected feed conversion ratio (MCFI:MCBWG); MCFI: mortality corrected feed intake.

2 CON: control commercial industry starter diet (n = 6 replicate pens) fed from d 0 to 14 posthatch; RED: 10% reduced digestible lysine (dLys) and metabolizable energy (ME) starter diet (n = 6 replicate pens) fed from d 0 to 14 posthatch.

3 SEM: largest standard error of the least square means.

ab Means within a row with different superscripts differ at *P* ≤ 0.05.

x,y Means within a row with different superscripts differ at 0.0501 < *P* ≤ 0.10.

**Figure 2:**
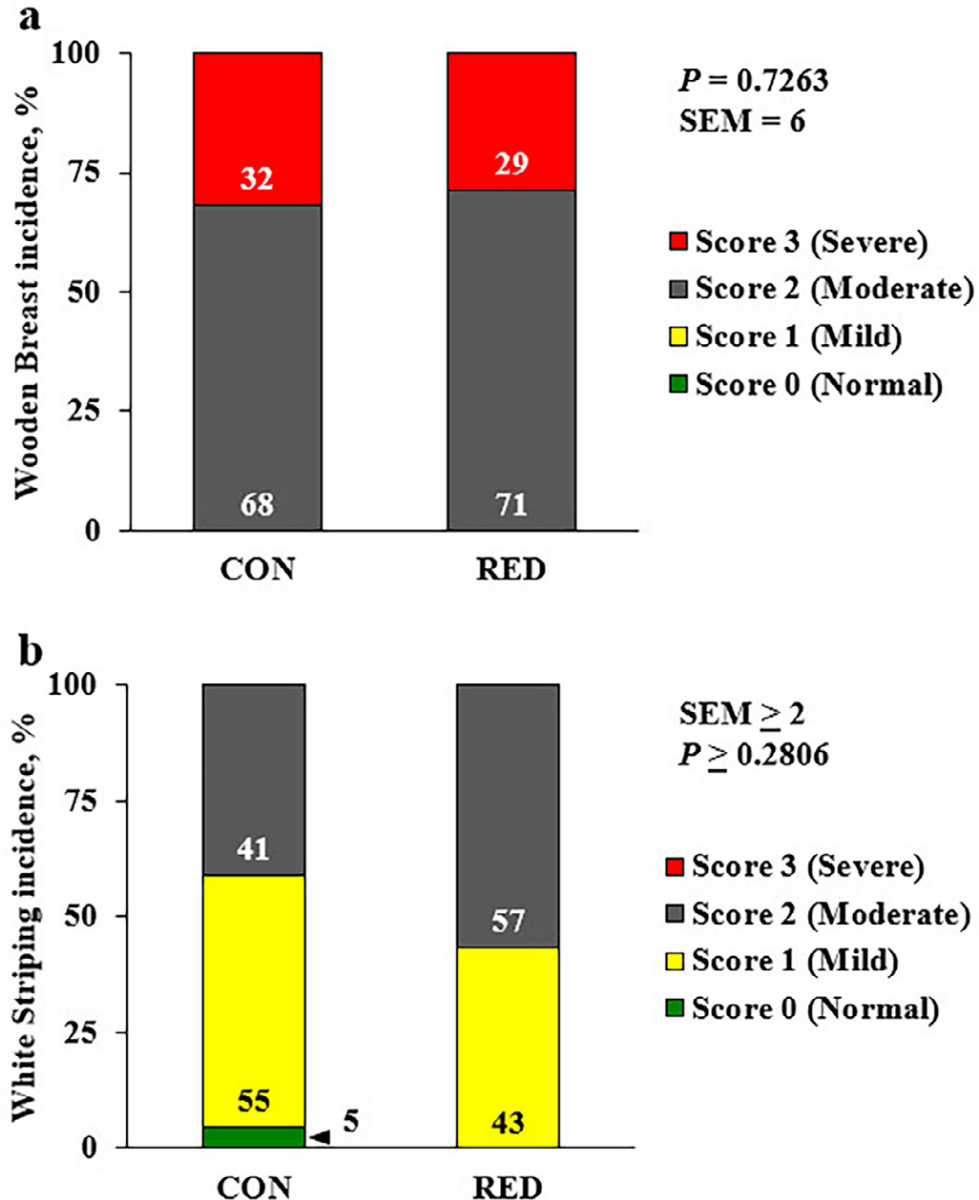
Effect of starter diet nutrient reduction on the incidence and severity of Wooden Breast and White Striping in broiler chickens at 36 d of age. Proportions of normal (0; green), mild (1; yellow), moderate (2; gray), and severe (3; red) scores for Wooden breast (A) and White striping (B) observed among male, Ross 708 × Yield Plus broiler chickens at 36 d of age. Broilers received 1 of 2 starter diets from d 0 to 14 posthatch in crumble form: a commercial industry control (CON; n = 22 sampled birds) starter diet or the CON diet with a 10% reduction in digestible lysine and metabolizable energy (RED; n = 28 sampled birds) from d 0 to 14 posthatch. All birds were fed a common commercial, pelleted grower diet from d 15 to 35. SEM, standard error of the mean.

Overall, the starter diet nutrient reduction had no effect on breast weight, breast meat yield, mean WB or WS scores (Table 5; *P* ≥ 0.4452), or the incidence and severity of WB or WS (Figures 2A and 2B; *P* > 0.2806). However, it is important to note that for WB neither treatment resulted in any normal (Score 0) or even mildly-affected (Score 1) breast fillets on d 35, but had 100% incidence of at least moderately (Score 2) or severely-affected (Score 3) breast fillets (Figure 2A). In terms of WS incidence and severity, neither starter diet treatment resulted in any severely-affected WS breast fillets on d 35 (Figure 2B). The vast majority of breast fillets scored among both treatments were at least mild or moderately affected with WS (Figure 2B). However, the CON treatment did produce 5% normal (Score 0) breast fillets, while the RED treatment resulted in no normal (Score 0) fillets (Figure 2B).

**Table 5:** The effect of starter diet nutrient reduction on broiler breast weight, breast meat yield, and mean Wooden Breast and White Striping scores on d 36.

	Starter diet^2^			
Variable^1^	CON	RED (10% ↓ dLys and ME)	SEM^3^	*P*-value
Breast weight, g	487	481	13	0.7454
Breast yield, %	18	18	0.3	0.7104
Mean WB score	2.3	2.3	0.1	0.8619
Mean WS score	1.4	1.6	0.1	0.4452

1 On an individual bird basis: Breast weight = total breast muscle weight grams; breast yield = breast meat weight as a percentage of body weight; mean WB score = mean Wooden Breast score; mean WS score = mean White Striping score.

2 CON = standard, control industry starter diet (n = 22 sampled birds) fed from d 0 to 14 posthatch; RED = 10% digestible lysine and metabolizable energy restricted starter diet (n = 28 sampled birds) fed from d 0 to 14 posthatch.

3 SEM = largest pooled standard error of the pairwise mean comparisons. ^ab^Means within row with different superscripts differ (*P* ≤ 0.05).

The increase in FI observed in the starter phase on the RED-fed birds indicates the birds simply consumed more feed to compensate for the 10% reduction in dLys and ME, resulting in compensatory gain during the grower (d 15–35) period as well as a tendency for greater BW on d 35. Compensatory gain in broiler chickens has been previously observed and well-reviewed (Zubair and Leeson, 1996). The findings of Leeson et al. (1991) revealed that a dilution in major nutrients in starter diets replaced by rice hulls reduced broiler BW at 11 d of age, but birds were able to compensate for the period of feed restriction once switched to a standard diet resulting in similar BW and carcass parts at 42 d of age. The data presented here are in agreement with the aforementioned study, demonstrating the ability of broilers to compensate for diet restrictions with increased BWG during the grower period, similar breast weights by the end of the study. Leeson et al. (1992) reported nutrient restrictions in finisher diets increased broiler FI, and thereby maintained BW and breast weights. In recent literature, Iwasaki and collaborators utilized a starter diet restriction for 12 d before switching all birds to a common finisher diet in meal form which yielded similar results to our study regarding compensatory gain when birds of the same sex were similar in BW by the end of the experiment (Iwasaki et al., 2021) even though this experiment utilized a common grower in pellet form after 14 d posthatch.

Simoes et al. (2020) previously demonstrated that slowing broiler growth rate by limiting intake to 90% or less of ad libitum intake can reduce WB incidence which is expected due to the high correlation of WB with growth rate (Petracci et al., 2019). Vieira et al. (2021) fed broilers a diet with low protein and energy density in different growth phases and found that early nutrient restriction reduced WB severity early on, but WB incidence was similar by d 49 after switching to common diets after 29 d posthatch. This indicates that dietary nutrient reduction may slow the progression of WB but does not prevent WB development in the long term. While in agreement with our work, these data contrast with a study by Meloche et al. (2018a) where a 15% targeted decrease in dLys in the grower 2 diet decreased the incidence and severity of WB at d 46. The differences among the bird genetic strains used, variation in the timing and specific type of the restrictions as well as the experimental end-points help explain the lack of congruence among the previous findings of other researchers and our work. Regardless, in the present experiment, a targeted 10% reduction in dLys and ME in the starter diet was not sufficient to produce reductions in the incidence and severity of WB or WS, which was the overall goal of this experiment.

## CONCLUSIONS

These results suggest that rearing birds under HIGHLI (30 lux) from d 0 to 35, causes birds to consume more feed in the starter period, and can improve broiler BW at d 35 and BWG during the grower phase compared with birds reared under LOWLI (2 lux). Feeding birds a starter diet from d 0 to 14 with a 10% targeted reduction in dLys and ME (RED) resulted in birds consuming more feed during that phase to compensate for the nutrient reduction in the diet, which allowed them to experience compensatory gain in the grower period (d 15–35) compared with birds fed a common, industry starter diet. Regardless of LI or starter diet offered, no differences were observed at d 36 in breast muscle weights, breast meat yield, or the incidence and severity of WB and WS. In conclusion, utilizing LI and starter diet nutrient reduction strategies to slow broiler growth and reduce the incidence and severity of WB did not prove a reliable strategy, and no interaction among these effects were observed in this study. To produce a flock of broilers that are differently affected by WB using starter diet nutrient reductions, it is likely that more extreme reductions in dLys content combined with reductions in ME may be required.

## DISCLOSURES

The authors declare that they have no known competing financial interests or personal relationships that could have appeared to influence the work reported in this paper.

## References

[bib0001] Avila L.P., Leiva S.F., Abascal-Ponciano G.A., Flees J.J., Sweeney K.M., Wilson J.L., Meloche K.J., Turner B.J., Litta G., Waguespack-Levy A.M., Pokoo-Aikins A., Starkey C.W., Starkey J.D. (2022). Effect of combined maternal and post-hatch dietary 25-hydroxycholecalciferol supplementation on broiler chicken *Pectoralis major* muscle growth characteristics and satellite cell mitotic activity. J. Anim. Sci..

[bib0002] Bodle B.C., Alvarado C., Shirley R.B., Mercier Y., Lee J.T. (2018). Evaluation of different dietary alterations in their ability to mitigate the incidence and severity of woody breast and white striping in commercial male broilers. Poult. Sci..

[bib0003] Chatterjee D., Zhuang H., Bowker B.C., Rincon A.M., Sanchez-Brambila G. (2016). Instrumental texture characteristics of broiler *Pectoralis major* with the wooden breast condition. Poult. Sci..

[bib0004] Cruz R.F., Vieira S.L., Kindlein L., Kipper M., Cemin H.S., Rauber S.M. (2017). Occurrence of white striping and wooden breast in broilers fed grower and finisher diets with increasing lysine levels. Poult. Sci..

[bib0005] de Almeida Assunção A.S., Garcia R.G., Komiyama C.M., de Sena Gandra É R., de Souza J.R., Dos Santos W., Caldara F.R., Martins R.A. (2020). Wooden breast myopathy on broiler breast fillets affects quality and consumer preference. Trop. Anim. Health Prod.

[bib0006] Deep A., Raginski C., Schwean-Lardner K., Fancher B.I., Classen H.L. (2013). Minimum light intensity threshold to prevent negative effects on broiler production and welfare. Br. Poult. Sci..

[bib0007] Ferreira T.Z., Kindlein L., Flees J.J., Shortnacy L.K., Vieira S.L., Nascimento V.P., Meloche K.J., Starkey J.D. (2020). Characterization of *Pectoralis major* muscle satellite cell population heterogeneity, macrophage density, and collagen infiltration in broiler chickens affected by wooden breast. Front. Physiol..

[bib0008] Iwasaki T., Watanabe T., Hasegawa Y., Hosotani M., Kawasaki T. (2021). Nutrition during the early rearing period affects the incidence of wooden breasts in broilers. J. Poult. Sci..

[bib0009] Kawasaki T., Iwasaki T., Yamada M., Yoshida T., Watanabe T. (2018). Rapid growth rate results in remarkably hardened breast in broilers during the middle stage of rearing: a biochemical and histopathological study. PLoS One.

[bib0010] Kim H.J., Son J., Kim H.S., Hong E.C., Kim J.H. (2022). Effects of light intensity on growth performance, blood components, carcass characteristics, and welfare of broilers. J. Anim. Sci. Technol..

[bib0011] Kuttappan V.A., Manangi M., Bekker M., Chen J., Vazquez-Anon M. (2021). nutritional intervention strategies using dietary antioxidants and organic trace minerals to reduce the incidence of wooden breast and other carcass quality defects in broiler birds. Front. Physiol..

[bib0012] Leeson A., Summers J.D., Caston L.J. (1991). Diet dilution and compensatory growth in broilers. Poult. Sci..

[bib0013] Leeson S., Summers J.D., Caston L.J. (1992). Response of broilers to feed restriction or diet dilution in the finisher period. Poult. Sci..

[bib0014] Lien R.J., Hess J.B., McKee S.R., Bilgili S.F. (2008). Effect of light intensity on live performance and processing characteristics of broilers. Poult. Sci..

[bib0015] Livingston M.L., Landon C., Barnes H.J., Brake J. (2019). White striping and wooden breast myopathies of broiler breast muscle is affected by time-limited feeding, genetic background, and egg storage. Poult. Sci..

[bib0016] Meloche K.J., Dozier W.A., Brandebourg T.D., Starkey J.D. (2018). Skeletal muscle growth characteristics and myogenic stem cell activity in broiler chickens affected by wooden breast. Poult. Sci..

[bib0017] Meloche K.J., Fancher B.I., Bilgili S.F., Emmerson D.A., Dozier W.A. (2016). Effects of reduced digestible lysine density on myopathies of the *Pectoralis major* muscles in broiler chickens at 46 d of age. Poult. Sci..

[bib0018] Meloche K.J., Fancher B.I., Emmerson D.A., Bilgili S.F., Dozier W.A. (2016). Effects of qualitative nutrient allocation on myopathies of the *Pectoralis major* muscles in broiler chickens at 48 d of age. Poult. Sci..

[bib0019] Meloche K.J., Fancher B.I., Emmerson D.A., Bilgili S.F., Dozier W.A. (2018). Effects of reduced dietary energy and amino acid density on *Pectoralis major* myopathies in broiler chickens at 36 and 49 days of age. Poult. Sci..

[bib0020] Meloche K.J., Fancher B.I., Emmerson D.A., Bilgili S.F., Dozier W.A. (2018). Effects of reduced digestible lysine density on myopathies of the *Pectoralis major* muscles in broiler chickens at 48 and 62 days of age. Poult. Sci..

[bib0021] Meloche K.J., Fancher B.I., Emmerson D.A., Bilgili S.F., Dozier W.A. (2018). Effects of quantitative nutrient allocation on myopathies of the *Pectoralis major* muscles in broiler chickens at 32, 43, and 50 days of age. Poult. Sci..

[bib0022] Mudalal S., Lorenzi M., Soglia F., Cavani C., Petracci M. (2015). Implications of white striping and wooden breast abnormalities on quality traits of raw and marinated chicken meat. Animal.

[bib0023] Petracci M., Soglia F., Madruga M., Carvalho L., Ida E., Estevez M. (2019). Wooden-breast, white striping, and spaghetti meat: causes, consequences and consumer perception of emerging broiler meat abnormalities. Compr. Rev. Food Sci. Food Saf..

[bib0024] Raccoursier M., Thaxton Y.V., Christensen K., Aldridge D.J., Scanes C.G. (2019). Light intensity preferences of broiler chickens: implications for welfare. Animal.

[bib0025] Rault J.L., Clark K., Groves P.J., Cronin G.M. (2017). Light intensity of 5 or 20 lux on broiler behavior, welfare and productivity. Poult. Sci..

[bib0026] Sihvo H.K., Immonen K., Puolanne E. (2014). Myodegeneration with fibrosis and regeneration in the *Pectoralis major* muscle of broilers. Vet. Pathol..

[bib0027] Simões C.T., Vieira S.L., Stefanello C., Kindlein L., Ferreira T., Favero A., Xavier B. (2020). An in vivo evaluation of the effects of feed restriction regimens on wooden breast using ultrasound images as a predictive tool. Br. Poult. Sci..

[bib0028] Soglia F., Mudalal S., Babini E., Di Nunzio M., Mazzoni M., Sirri F., Cavani C., Petracci M. (2016). Histology, composition, and quality traits of chicken *Pectoralis major* muscle affected by wooden breast abnormality. Poult. Sci..

[bib0029] Tejeda O.J., Meloche K.J., Starkey J.D. (2021). Effect of incubator tray location on broiler chicken growth performance, carcass part yields, and the meat quality defects wooden breast and white striping. Poult. Sci..

[bib0030] Velleman S.G. (2020). *Pectoralis major* (breast) muscle extracellular matrix fibrillar collagen modifications associated with the wooden breast fibrotic myopathy in broilers. Front. Physiol..

[bib0031] Velo R., Ceular A. (2017). Effects of stocking density, light and perches on broiler growth. Anim. Sci. J..

[bib0032] Vieira S.L., Simoes C.T., Kindlein L., Ferreira T.Z., Soster P., Stefanello C. (2021). Progressive in vivo detection of wooden breast in broilers as affected by dietary energy and protein. Poult. Sci..

[bib0033] Wang C., Che S., Susta L., Barbut S. (2023). Textural and physical properties of breast fillets with myopathies (wooden breast, white striping, spaghetti meat) in Canadian fast-growing broiler chickens. Poult. Sci..

[bib0034] Wang J., Clark D.L., Jacobi S.K., Velleman S.G. (2020). Effect of early posthatch supplementation of vitamin E and omega-3 fatty acids on the severity of wooden breast, breast muscle morphological structure, and gene expression in the broiler breast muscle. Poult. Sci..

[bib0035] Zampiga M., Soglia F., Petracci M., Meluzzi A., Sirri F. (2019). Effect of different arginine-to-lysine ratios in broiler chicken diets on the occurrence of breast myopathies and meat quality attributes. Poult. Sci..

[bib0036] Zubair A., Leeson S. (1996). Compensatory growth in the broiler chicken: a review. Worlds Poult. Sci. J..

[bib0037] Zuidhof M.J., Schneider B.L., Carney V.L., Korver D.R., Robinson F.E. (2014). Growth, efficiency, and yield of commercial broilers from 1957, 1978, and 2005. Poult. Sci..

